# Room-Temperature CH_4_ Detection Using PLD-Deposited ZnO/CuO and ZnO/In_2_O_3_ Bilayer SAW Sensors

**DOI:** 10.3390/nano16211390

**Published:** 2026-10-29

**Authors:** Izabela Constantinoiu, Cristian Viespe, Cornelia Enache

**Affiliations:** Laser Department, National Institute for Laser, Plasma and Radiation Physics, Atomistilor 409, 077125 Magurele, Romania; izabela.constantinoiu@inflpr.ro (I.C.); cristian.viespe@inflpr.ro (C.V.)

**Keywords:** laser ablation, zinc oxide, copper oxide, indium oxide, methane, room-temperature gas sensing, surface acoustic wave sensor

## Abstract

This study investigates ZnO-based surface acoustic wave (SAW) sensors for the detection of methane (CH_4_) at room temperature (RT), focusing on the influence of film morphology and oxide heterostructures developed by pulsed laser deposition (PLD). Bilayer thin films—specifically ZnO/CuO and ZnO/In_2_O_3_—were deposited onto quartz substrates at 400 °C under oxygen pressures of 100 and 700 mTorr to evaluate how deposition conditions affect sensor performance. Structural and morphological characterization was performed using X-ray diffraction (XRD), scanning electron microscopy (SEM), and energy-dispersive X-ray spectroscopy (EDS). ZnO thin films deposited at 700 mTorr exhibited a more open, granular morphology compared to those deposited at 100 mTorr, a feature likely to facilitate gas access to the sensor’s active surface. The addition of CuO and In_2_O_3_ top layers further influenced the sensor response, presumably through surface and interface effects. Among the configurations studied, sensor S6 (ZnO/CuO, with both layers deposited at 700 mTorr) demonstrated the highest sensitivity—0.18 Hz/ppm—within the experimentally investigated CH_4_ concentration range (0.4–2%), with an estimated limit of detection (LOD) of 167 ppm. Short-term measurements performed on sensors S4 and S6 at a 2% CH_4_ concentration indicated reversible responses over two consecutive exposure and recovery cycles, with a return to the initial baseline value following the removal of the gas. These results suggest that ZnO-based double layers, fabricated via PLD, are promising candidates for further investigation as CH_4_-sensitive layers at RT.

## 1. Introduction

Methane (CH_4_), the primary constituent of natural gas, is widely used as an energy source, but it also raises important environmental and safety concerns. CH_4_ emissions from fossil fuel extraction, transport, and use contribute to greenhouse gas accumulation, while CH_4_ leaks are difficult to detect directly because the gas is colorless and odorless. Accurate and rapid CH_4_ detection is therefore important for industrial safety, distributed energy infrastructure, and environmental monitoring [1,2].

A broad range of CH_4_-sensing technologies has been explored, including optical, semiconductor, and acoustic platforms, but room-temperature (RT) operation remains difficult because CH_4_ is relatively inert and interacts weakly with many sensing materials [1,3]. Conventional metal oxide sensors often require elevated temperatures above 200 °C, which increases power consumption and limits practical deployment [3,4]. Even so, recent work shows that RT CH_4_ sensing is feasible when the sensing layer and transducer are carefully engineered [1,3,5].

Among gas-sensing technologies, surface acoustic wave (SAW) sensors are attractive because they combine high sensitivity, compact size, low cost, and the possibility of wireless or passive operation [6,7,8]. A typical SAW sensor contains interdigital transducers on a piezoelectric substrate and a sensing coating deposited in the delay path; gas adsorption changes film mass, conductivity, or elasticity, which shifts acoustic wave velocity and frequency [6,9]. Because the bare SAW device is not intrinsically selective, the material and structure of the sensing layer largely determine the sensitivity, selectivity, reversibility, and response kinetics of the sensor [6,8].

For CH_4_ in particular, SAW sensors are promising for ambient-condition monitoring, but practical performance still depends strongly on the design of the sensitive layer [6]. Previous CH_4_-specific SAW studies have shown that the frequency shift can be correlated with CH_4_ concentration and that RT operation is achievable, although detection limits and cross-sensitivity remain important challenges [3,6].

Among oxide semiconductors used in acoustic and gas-sensing devices, ZnO is especially attractive because it is a wide-band-gap *n*-type semiconductor with useful piezoelectric, chemical, thermal and sensing properties [4]. ZnO is already well established for gas sensing, but most ZnO gas sensors still perform best at higher operating temperatures rather than at RT [4,10]. In SAW platforms, ZnO is additionally valuable because adsorbed species can modify its surface conductivity, enabling both mass-loading and acoustoelectric contributions to the sensing response [6].

The sensing performance of ZnO depends strongly on morphology, porosity, thickness, crystallinity, and defect structure [11,12]. A porous, open granular morphology and nanostructured ZnO layers generally improve gas response because they provide a larger accessible surface area and more efficient diffusion pathways [4,13,14]. This dependence is evident across both acoustic and non-acoustic systems: nanorod-type ZnO can outperform more compact or nanograined morphologies, while film thickness can also influence sensitivity by changing available adsorption volume [11].

A second effective strategy is to combine ZnO with another oxide to obtain a heterostructure, since heterogeneous interfaces can increase surface area, oxygen-vacancy density, internal electric field strength, and charge-transfer efficiency [13,14,15]. Bilayers and composite sensing films can shift the operating point into a more sensitive regime, because interfacial conductivity changes are amplified by the heterojunction [5,16]. This logic is relevant for CuO and In_2_O_3_ modification of ZnO because these oxides alter adsorption behavior and interfacial charge transport in different ways. CuO can form a *p*-type counterpart to *n*-type ZnO, while indium-containing ZnO heterostructures have already shown RT CH_4_ sensing in other platforms [1].

Pulsed laser deposition (PLD) is well suited to fabricate such oxide films because it offers broad control over thickness, crystallinity, surface morphology, and stoichiometry through parameters such as oxygen pressure, laser fluence, pulse number, and substrate temperature [17,18]. Across PLD-grown ZnO films, changing deposition conditions alters orientation, defect population, particle size, and porosity, and these structural differences translate into measurable changes in gas-sensing behavior [17,18]. Higher background pressure in PLD tends to increase collisions among ablated species and can promote the formation of smaller building units and more porous films or a more open granular morphology, which has been linked to improved sensor response [18,19,20].

The present study compares ZnO, ZnO/CuO, and ZnO/In_2_O_3_ films deposited by PLD as sensing layers for RT CH_4_ detection using SAW devices. The objective is to examine how the oxygen pressure used for ZnO deposition and the addition of oxide overlayers affect the measured frequency response. Structural and morphological observations are used to discuss possible relationships between film characteristics and sensor response; the proposed surface and interfacial mechanisms are distinguished from directly measured results.

## 2. Materials and Methods

### 2.1. Thin Film Deposition

Thin films were deposited using a PLD system, utilizing an experimental setup described in detail in [21]. A Nd:YAG laser (EKSPLA NL301HT, Ekspla, Vilnius, Lithuania) operating at a wavelength of 355 nm, with a pulse duration of 5 ns and a repetition rate of 10 Hz, was employed to ablate the target materials. The laser beam was focused onto the target surface at a fluence of 0.54 J/cm^2^, generating a plasma plume of ablated species. The deposition process was carried out in a high-vacuum chamber equipped with a gas pressure controller (MKS 600, Munchen, Germany) and a mass-flow control system (MKS multigas 647, Munchen, Germany) (base pressure of 10^−5^ mbar) to ensure minimal background contamination, after which high-purity oxygen (99.999%) was introduced to serve as the reactive background gas. The ablated species were subsequently transported through the oxygen atmosphere and deposited onto the substrates, leading to the formation of the oxide thin films.

Prior to deposition, the quartz substrates were ultrasonically cleaned in alcohol for approximately 15 min to remove surface contaminants and ensure optimal film adhesion. The target-to-substrate distance was maintained at 40 mm, and the substrate temperature was fixed at 400 °C. To investigate the influence of deposition pressure on the structural and sensing properties, the films were grown under two distinct oxygen background pressures: 100 mTorr and 700 mTorr. After the deposition process was completed, the samples were cooled to RT within the chamber under the same oxygen atmosphere. The targets were placed on a computer-controlled X-Y translation stage, operated using a custom-developed control program, to ensure uniform laser ablation across the target surface and prevent localized target erosion.

Three distinct configurations were synthesized for this study, bare ZnO films, ZnO/CuO bilayers, and ZnO/In_2_O_3_ bilayers, as summarized in Table 1. The bare ZnO films were deposited directly from a ZnO target using 36,000 laser pulses. The CuO and In_2_O_3_ overlayers were deposited starting from pure metallic copper (Cu) and indium (In) targets, respectively, reacting in situ with the oxygen atmosphere to form the corresponding oxides; these overlayers were grown using 1800 laser pulses. This approach allowed for the systematic study of the influence of CuO and In_2_O_3_ functionalization on the SAW sensor performance.

The crystalline structure and morphological properties of the deposited films were investigated by X-ray diffraction (XRD) and scanning electron microscopy (SEM). XRD measurements were collected using a PANalytical X’Pert Pro MPD diffractometer (PANalytical B.V., Almelo, The Netherlands) (2.2 kW, 60 kV; λ = 1.5406 Å, CuKα radiation). Surface morphology was examined using an FEI Inspect S SEM (Hillsboro, OR, USA) equipped with a field emission gun, coupled with an energy-dispersive X-ray spectroscopy (EDS) system for compositional analysis of the deposited films.

### 2.2. Sensor Configuration

The SAW sensors (Figure 1) were fabricated on ST-X cut quartz piezoelectric substrates, selected for their stable temperature coefficient, with a parallelogram geometry (38 mm × 10 mm × 0.5 mm) with 45° angle to reduce unwanted SAW reflections. Two pairs of gold interdigitated transducers (IDTs) were deposited by photolithography onto a 60 nm chromium adhesion layer, with a gold thickness of ~150 nm. Each double-comb transducer comprised 50 finger pairs, and the sensing layer was positioned between the two IDT pairs. The device operated as a delay-line sensor at ~65 MHz with a parallelogram geometry to minimize wave reflection effects. An electrical signal applied to the input IDT was converted into a SAW by the piezoelectric quartz, propagated across the sensing layer, and reconverted into an electrical signal at the output IDT. Changes in the sensing layer properties altered the wave oscillation frequency, generating a sensor signal proportional to the analyte concentration.

### 2.3. Gas Testing Setup

Gas-sensing measurements were collected using a mass flow controller (MKS Instruments, MKS multigas 647, Munchen, Germany) that mixed 2% CH_4_ in synthetic air with 100% synthetic air to achieve the desired CH_4_ concentrations. The total gas flow was maintained constant at 0.5 L/min throughout all measurements to eliminate flow-induced frequency shifts. The oscillation circuit (Figure 1) included the SAW device, a phase shifter (PSV-70-360-S; IF Engineering Corp., North Grosvenordale, CT, USA), a bandpass filter (Anatech Electronics B9336, Inc., Garfield, NJ, USA), and an amplifier (DHPVA-100 FEMTO, Messtechnik GmbH, Berlin, Germany). Frequency shifts were recorded with a Pendulum CNT-91 Timer/Counter/Analyzer (Spectracom Corp, Rochester, NY, USA) using TimeView 2.1 software (Pendulum Instruments, Banino, Poland). All components were impedance-matched to 50 Ω using appropriate inductors, with signal attenuation and phase characteristics determined via a network/spectrum/impedance analyzer (4396B; Agilent Technologies, Santa Clara, CA, USA) equipped with a transmission/reflection kit (4396B; Agilent Technologies, Santa Clara, CA, USA).

## 3. Results and Discussion

### 3.1. Thin Film Characterization

The XRD patterns in Figure 2 and Figure 3 show reflections assigned to wurtzite ZnO (ICDD 00-036-1451). The additional reflection in the In-containing samples is assigned to cubic In_2_O_3_ (ICDD 00-006-0416). In the Cu-containing samples, the feature near 72–73° is compatible with CuO (311), but it overlaps with the region of the ZnO (004) reflection. This feature alone therefore does not provide an unambiguous identification of CuO. The phase assignments should be considered within the detection and peak-overlap limitations of the measurements. EDS analysis supports the presence of Cu and In but does not independently determine their oxidation states. Similar behavior has been widely reported for copper and indium thin films processed in oxygen-containing atmospheres, where XRD confirms the formation of CuO and In_2_O_3_ crystalline phases after oxidation or reactive deposition [22,23,24,25]. Therefore, in the present samples, the variation in oxygen pressure is more likely to affect crystallinity, crystallite size, defect density, and morphology than the basic phase composition of the films [22,23].

The crystallite sizes of the ZnO, CuO, and In_2_O_3_ thin films were estimated from the XRD patterns using the Scherrer Equation (1) [26]:

(1) $$D=\frac{K\cdot \lambda }{\beta \cdot cos\theta }$$

where *D* represents the estimated crystallite size, *K* is the dimensionless shape factor, taken as 0.9, and *λ* is the wavelength of the Cu Kα radiation (1.5406 Å). The parameter *β* represents the full width at half maximum (FWHM) of the selected diffraction peak, expressed in radians. FWHM describes the angular width of a diffraction peak measured at half of its maximum intensity above the local background. The parameter *θ* is the Bragg angle, corresponding to half of the measured diffraction angle (2*θ*).

The estimated apparent crystallite sizes were 26.3 nm and 24.7 nm for ZnO films deposited at oxygen pressures of 100 mTorr and 700 mTorr, respectively. For CuO films, the corresponding value was 21.1 nm, while the In_2_O_3_ films showed a value of 22.6 nm at 700 mTorr. These results suggest a slight decrease in the apparent crystallite size of ZnO as the oxygen pressure increases.

Since no correction for instrumental line broadening was applied, the calculated values should be considered estimates of the apparent sizes of the coherent diffraction domains. The results may also be influenced by contributions from microstrains and structural defects, which cause broadening of the peaks [27].

The SEM images (Figure 4) show a comparatively compact surface for ZnO deposited at 100 mTorr and a more open granular appearance at 700 mTorr. This qualitative difference may affect the accessibility of the sensing layer to the gas. However, SEM appearance alone does not quantify porosity or specific surface area.

The film deposited at 100 mTorr (Figure 4A) shows a more compact and continuous morphology, whereas the film grown at 700 mTorr (Figure 4B) exhibits a more open granular morphology, with well-defined grains separated by intergranular voids, indicating a less densely packed microstructure. This trend is consistent with previous reports showing that oxygen partial pressure strongly affects ZnO surface morphology, packing density, grain development, and roughness [28,29,30,31]. In ZnO systems, lower oxygen pressure is commonly associated with denser or more continuous films, while increasing the oxygen pressure can promote rougher, more granular, or more open morphologies because of enhanced gas-phase collisions and reduced kinetic energy of the arriving species [28,29]. Therefore, in the present case, the more open granular morphology observed at 700 mTorr is reasonably attributed to pressure-assisted modification of the ZnO growth regime, which favors the formation of a more accessible surface area, a feature that is often beneficial for gas-sensing applications [28,32,33].

For SAW sensors, the sensitive film must provide not only a sufficiently open granular morphology for efficient gas adsorption and diffusion, but also good adhesion and structural stability on the device surface [34,35,36]. In general, higher porosity improves gas access and can enhance sensor response, whereas film uniformity, compactness, and mechanical and chemical robustness are essential for reproducible and durable operation [34,37,38]. Therefore, the sensing layer should achieve a balance between these characteristics, since an excessively dense film may limit analyte access, while an overly loose or weakly adhered film can compromise acoustic performance and long-term stability.

The EDS maps (Figure 5 and Figure 6) show Zn, Cu or In, and O distributed across the analyzed areas. No obvious large-scale segregation is apparent at the resolution of these maps. The Si signal is consistent with a contribution from the quartz substrate, which may also contribute to the oxygen signal. These measurements support the presence and lateral distribution of the detected elements.

In the ZnO/CuO film, Zn, Cu, and O appear broadly distributed across the analyzed area, without obvious micron-scale segregation at the resolution of the EDS maps. In the ZnO/In_2_O_3_ film, Zn, In, and O similarly show uniform distributions, consistent with good compositional homogeneity of the heterostructure. These results support the elemental homogeneity of the deposited bilayers, which is important for achieving uniform sensing properties across the active SAW area.

### 3.2. Sensor Characterization

Sensor testing at CH_4_ concentrations from 0.4% to 2% revealed a distinct performance hierarchy that may be associated with differences in morphology and interfacial effects.

The highest frequency shifts (Figure 7) were recorded for S6 (ZnO/CuO, both at 700 mTorr), followed by S4 (ZnO/In_2_O_3_, both at 700 mTorr), and S2 (ZnO at 700 mTorr). A significant performance drop separated these from the remaining sensors: S5 (ZnO at 100 mTorr/CuO at 700 mTorr), S3 (ZnO at 100 mTorr/In_2_O_3_ at 700 mTorr), and S1 (bare ZnO at 100 mTorr). This reported response ranking was consistently reflected in the calculated sensitivity and limit of detection (LOD) values, presented in Table 2. The frequency shifts in Figure 7 were calculated relative to the baseline frequency before CH_4_ exposure, using the frequency recorded approximately 320 s after the onset of the sensor response.

Sensitivity was expressed as the change in frequency per unit of CH_4_ concentration (Hz/ppm). The baseline frequency fluctuations were evaluated over a 10 min period under a constant synthetic air flow of 0.5 L/min, in the absence of CH_4_. The maximum absolute deviation from the mean frequency was approximately 10 Hz. This value was used as a conservative noise estimate (*n*) to calculate the estimated limit of detection (LOD) using the relationship LOD = 3*n*/S, where S is the calculated sensitivity. The estimated LOD values were derived from responses measured within the investigated concentration range of 4000–20,000 ppm CH_4_. For sensor S6, the estimated LOD value of 167 ppm (Table 2) is significantly lower than the minimum concentration tested experimentally (4000 ppm). Consequently, this value represents a theoretical estimate based on experimental data.

Differences in the responses measured for CH_4_ may be attributed to variations in the ZnO surface morphology and the presence of additional oxide layers. SEM observations indicate that ZnO deposited at 700 mTorr exhibits a more open, granular morphology compared to films deposited at 100 mTorr; this may facilitate gas access into the bulk of the sensor’s sensitive film. The addition of CuO and In_2_O_3_ layers can also influence surface interactions and charge transport at the interface, contributing to the larger frequency shifts observed in sensors with a layered structure.

Table 3 compares the CH_4_ detection performance of the SAW-type sensors developed in this work with that of previously reported devices that utilize different sensing materials. Several studies have demonstrated CH_4_ detection at RT with relatively low detection limits, although the concentration ranges investigated, the sensing materials used, and the experimental conditions vary considerably. In the present study, the ZnO/CuO double-layer sensor (S6) exhibited the best performance, with an estimated detection limit (LOD) of 167 ppm. Although lower detection limits have been reported for other sensing materials, the current results highlight the potential of oxide double layers, deposited via the PLD technique, for RT CH_4_ detection. Further investigations into response/recovery times, the effects of humidity, and selectivity are required for a more comprehensive comparison of performance.

For ZnO, ZnO/CuO, and ZnO/In_2_O_3_ films, the proposed sensing mechanism involves surface adsorption and reactions, as well as charge transfer at the heterojunction interfaces. On ZnO, oxygen first adsorbs from air and traps electrons at the surface, creating chemisorbed oxygen species and a depleted surface layer before CH_4_ is introduced [42,43]. Furthermore, the state of oxygen on the surface of metal oxides can influence gas-sensing performance by affecting the availability of adsorption sites and surface charge-transfer processes. Recent studies on oxide-based heterostructures have also highlighted the importance of surface chemistry and interfacial interactions in enhancing the gas-sensing response [44]. However, surface oxygen species were not directly investigated in this study, and their specific contribution to the response toward CH_4_ remains to be clarified.

CH_4_ is a weak reducing gas, so its direct interaction is limited unless reactive oxygen and active sites are already present on the surface.

The response of metal oxide-based SAW sensors may involve several contributions, including gas adsorption and changes in the electrical conductivity, mass, and elastic properties of the sensing layer. Surface oxygen species may also influence gas-sensing behavior by affecting adsorption processes and surface charge transfer. Previous studies have discussed the interaction of CH_4_ with adsorbed oxygen species on ZnO surfaces [42,43]. However, Duangmanee et al. [43] investigated methane sensing at elevated temperatures, reporting optimal sensing behavior at approximately 300–350 °C. Therefore, the reaction mechanisms described in that study cannot be directly extrapolated to the RT conditions investigated here.

A further possible contribution is signal amplification at the interface. In oxide heterostructures, differences in Fermi level drive interfacial charge transfer and band bending, forming depletion or accumulation regions and a potential barrier that is highly sensitive to surface reactions [42]. Reviews consistently describe the benefit of heterojunctions as a combination of higher adsorption/reactivity and stronger transduction of that chemistry into an electrical signal [42].

For ZnO/CuO, the formation of a *p*–n junction could enhance surface reactivity and modulate charge transport. CuO addition may increase the availability of reactive sites and the defect density, while charge transfer at ZnO/CuO interfaces could contribute to a larger response than that of bare ZnO. In heterojunction systems containing In_2_O_3_ and CuO, CH_4_ adsorption may modify interfacial electronic properties and thereby contribute to the detected signal.

The ZnO layer deposited at 700 mTorr exhibits a more open granular morphology than that deposited at 100 mTorr, which may improve gas accessibility and facilitate CH_4_ adsorption and diffusion through the active layer. This could help explain why all sensors based on ZnO deposited at 700 mTorr outperformed the corresponding 100 mTorr version.

The performance order S6 > S4 > S2 may reflect contributions from interfacial effects. If a CuO/ZnO *p*-n junction is present, depletion-layer modulation and oxygen-vacancy-assisted charge transfer could contribute to the enhanced response [45], while additional reactive sites and interfacial barriers may also play a role [42,46].

The response of bare ZnO deposited at 700 mTorr may benefit from its more open granular morphology, which could facilitate CH_4_ adsorption and oxygen-mediated electron exchange. The lower responses of S5, S3 and S1 may partly be explained by the denser ZnO sublayer deposited at 100 mTorr, which could limit gas access to reactive sites and interfacial regions.

Figure 8 and Figure 9 show the dynamic frequency responses of sensors S4 and S6, respectively, over two consecutive cycles of exposure to CH_4_ and subsequent recovery, at a concentration of 2%. Each CH_4_ exposure lasted approximately 350–370 s. Both sensors exhibited reversible frequency variations and returned to their initial baseline values once the CH_4_ was removed. These observations indicate reversible sensing behavior in the short term under the conditions investigated.

Although the sensors demonstrated good short-term repeatability, their long-term stability remains to be investigated through systematic aging tests—including storage for a period of 7–14 days—as well as extended monitoring of baseline drift, sensitivity, and response/recovery characteristics.

While the developed SAW-based sensors showed measurable responses to CH_4_ at RT, their selectivity toward other gases has not been investigated. Since SAW devices can also respond to other gaseous species through changes in the properties of the active layer, potential interference from gases such as CO, H_2_, and volatile organic compounds—as well as variations in humidity—cannot be ruled out. Consequently, these sensors should be regarded as preliminary platforms for CH_4_ monitoring under controlled conditions.

Overall, the observed response trends are consistent with the hypothesis that a less densely packed granular ZnO surface facilitates gas access and surface interactions, while CuO- and In_2_O_3_-related interfacial effects may contribute to larger frequency shifts.

## 4. Conclusions

This study investigated the CH_4_ detection performance of SAW sensors at RT, utilizing sensitive thin layers of ZnO, ZnO/CuO, and ZnO/In_2_O_3_ fabricated via the PLD technique. The results indicate that the oxygen pressure employed during ZnO deposition influences both the layer morphology and the sensor response to CH_4_. ZnO deposited at a pressure of 700 mTorr exhibited a more open, granular morphology and greater frequency shifts upon exposure to CH_4_ compared to layers deposited at 100 mTorr. The addition of CuO and In_2_O_3_ overlayers further modified the sensing behavior, with the ZnO/CuO configuration (S6) demonstrating the highest sensitivity—0.18 Hz/ppm—among the devices investigated. The estimated limit of detection (LOD) for S6 was 167 ppm, calculated based on the measured sensitivity and frequency noise. Short-term exposure and recovery measurements performed on sensors S4 and S6 showed reversible frequency responses over two consecutive cycles at a 2% CH_4_ concentration. Further measurements are required to evaluate repeatability over a larger number of cycles, as well as long-term stability. Overall, the results demonstrate the influence of PLD conditions and dual-oxide layer configurations on the RT CH_4_ response of SAW sensors.

## Figures and Tables

**Figure 1:**
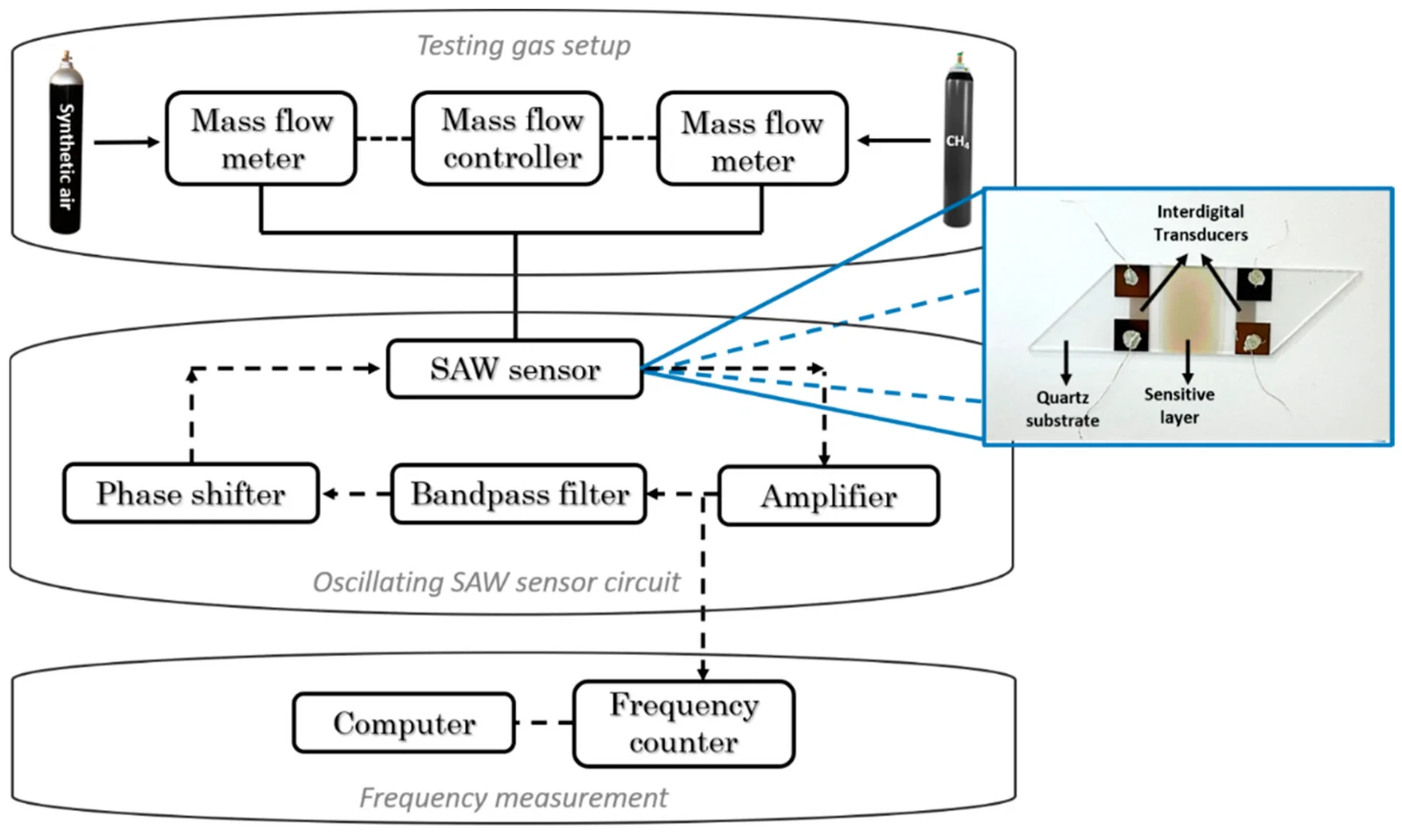
Oscillating circuit used for SAW sensor measurements.

**Figure 2:**
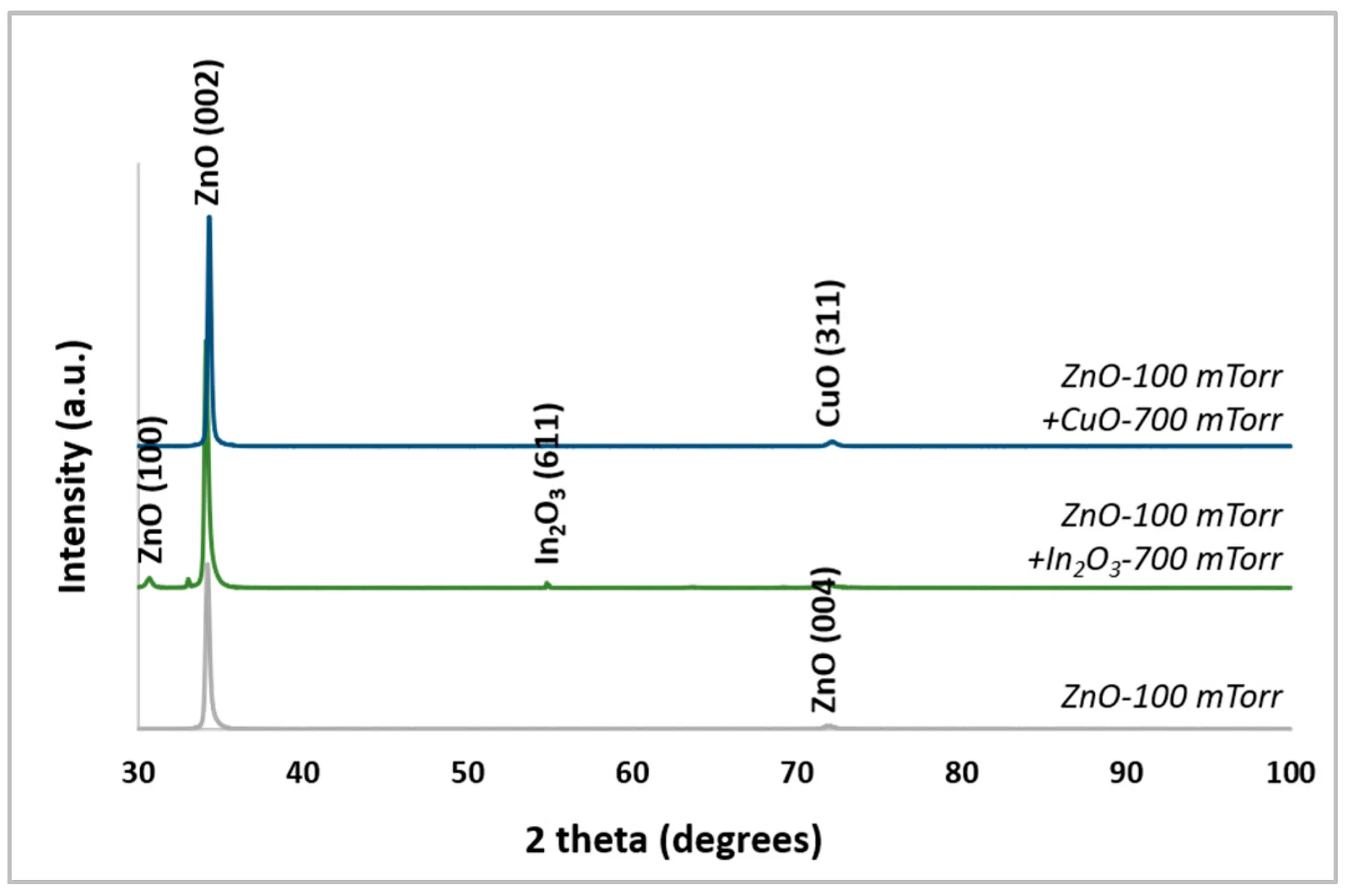
XRD analysis of the samples with ZnO films deposited at 100 mTorr.

**Figure 3:**
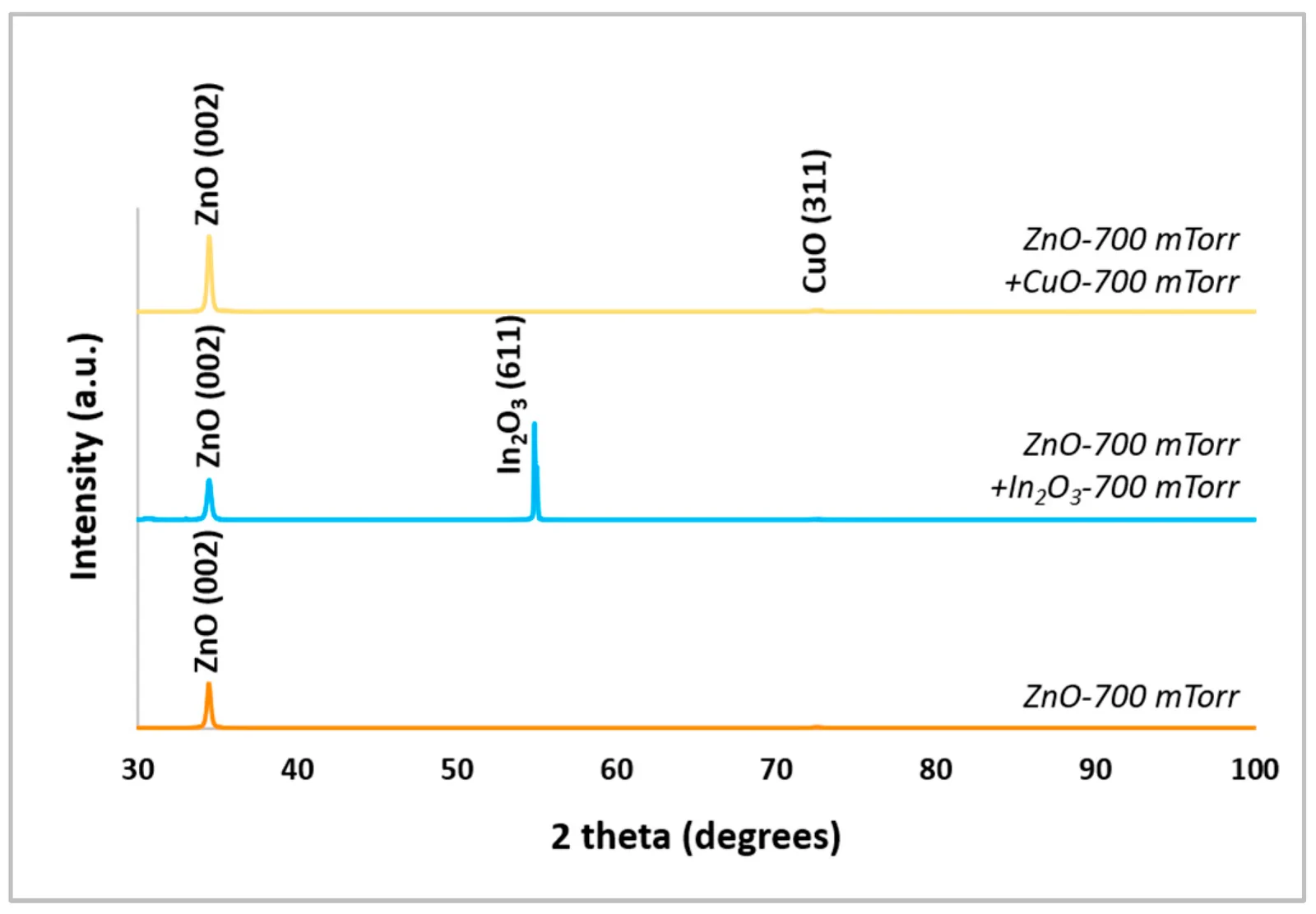
XRD analysis of the samples with ZnO films deposited at 700 mTorr.

**Figure 4:**
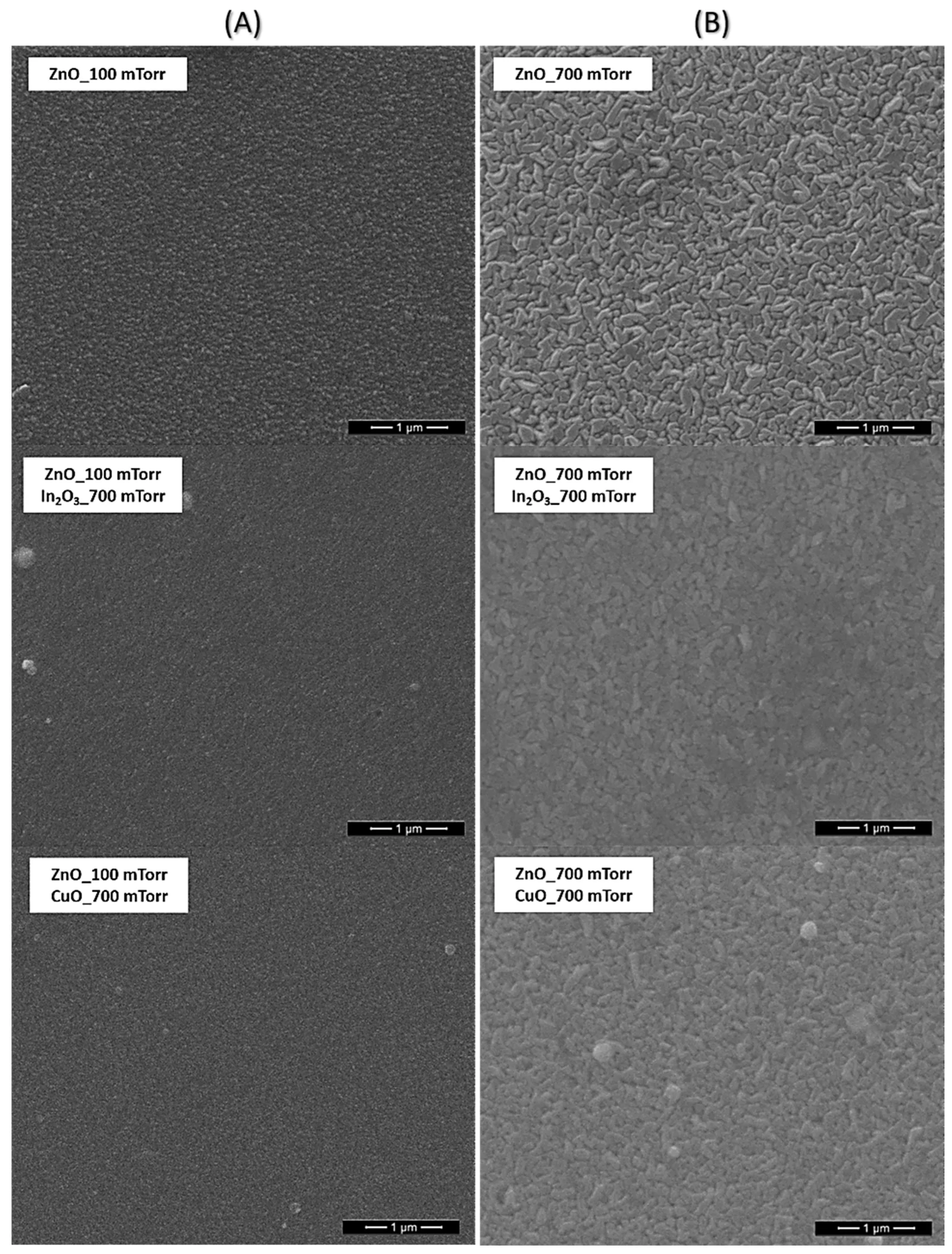
SEM images of the samples with ZnO films deposited at (**A**) 100 and (**B**) 700 mTorr.

**Figure 5:**
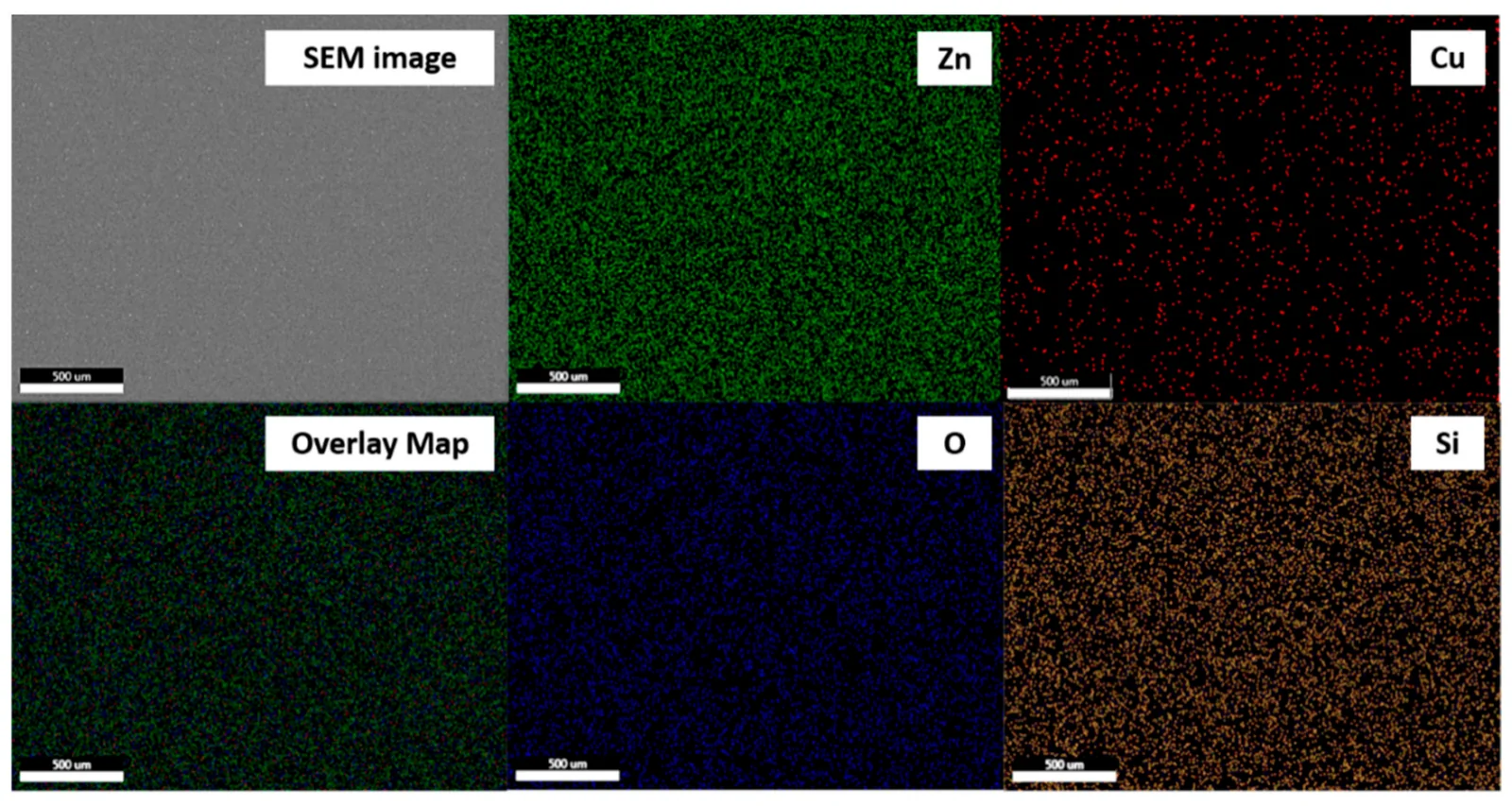
EDS elemental maps of the ZnO and CuO films deposited at 700 mTorr.

**Figure 6:**
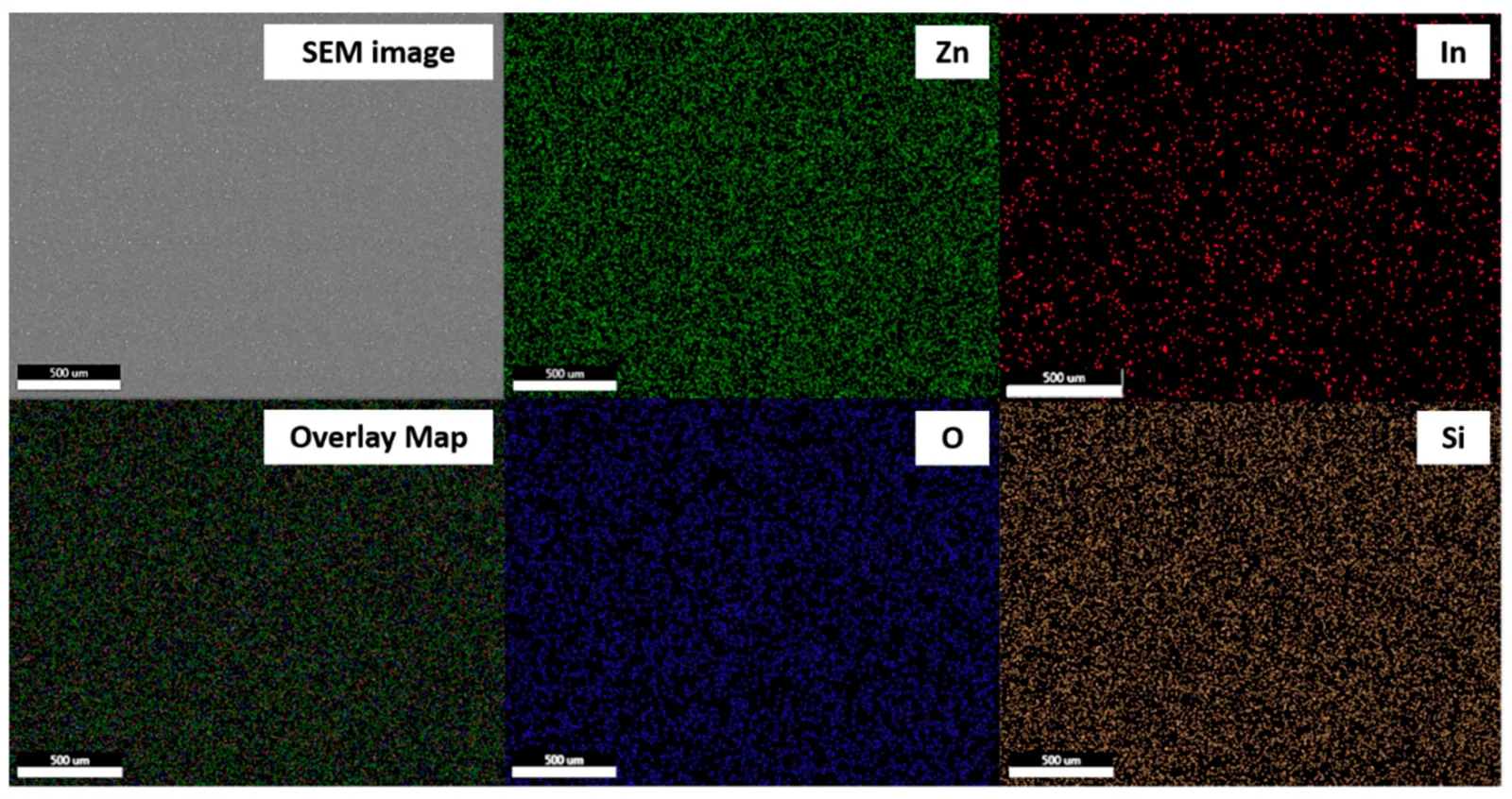
EDS elemental maps of the ZnO and In_2_O_3_ films deposited at 700 mTorr.

**Figure 7:**
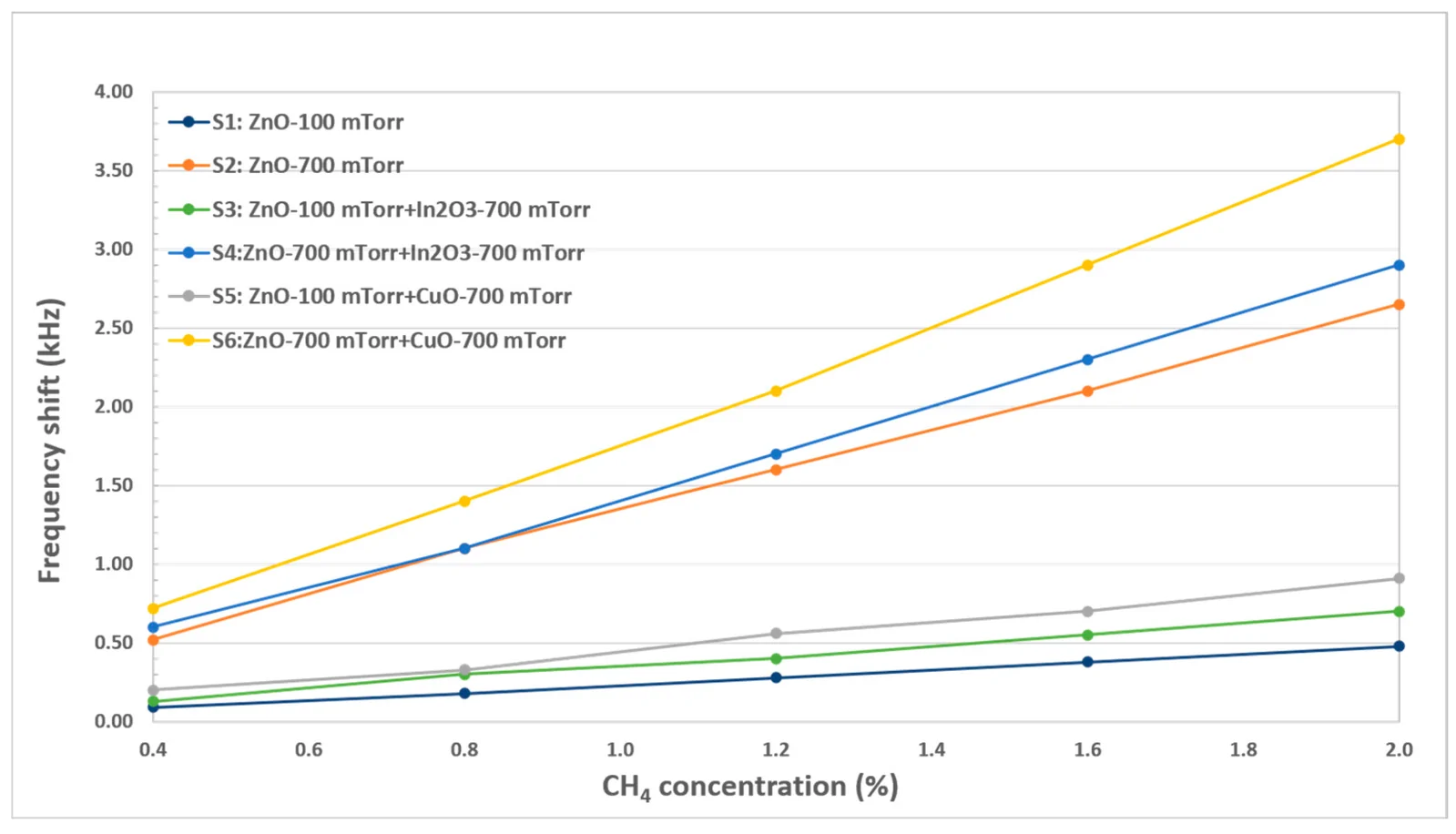
Frequency shift in the SAW sensors at different CH_4_ concentrations.

**Figure 8:**
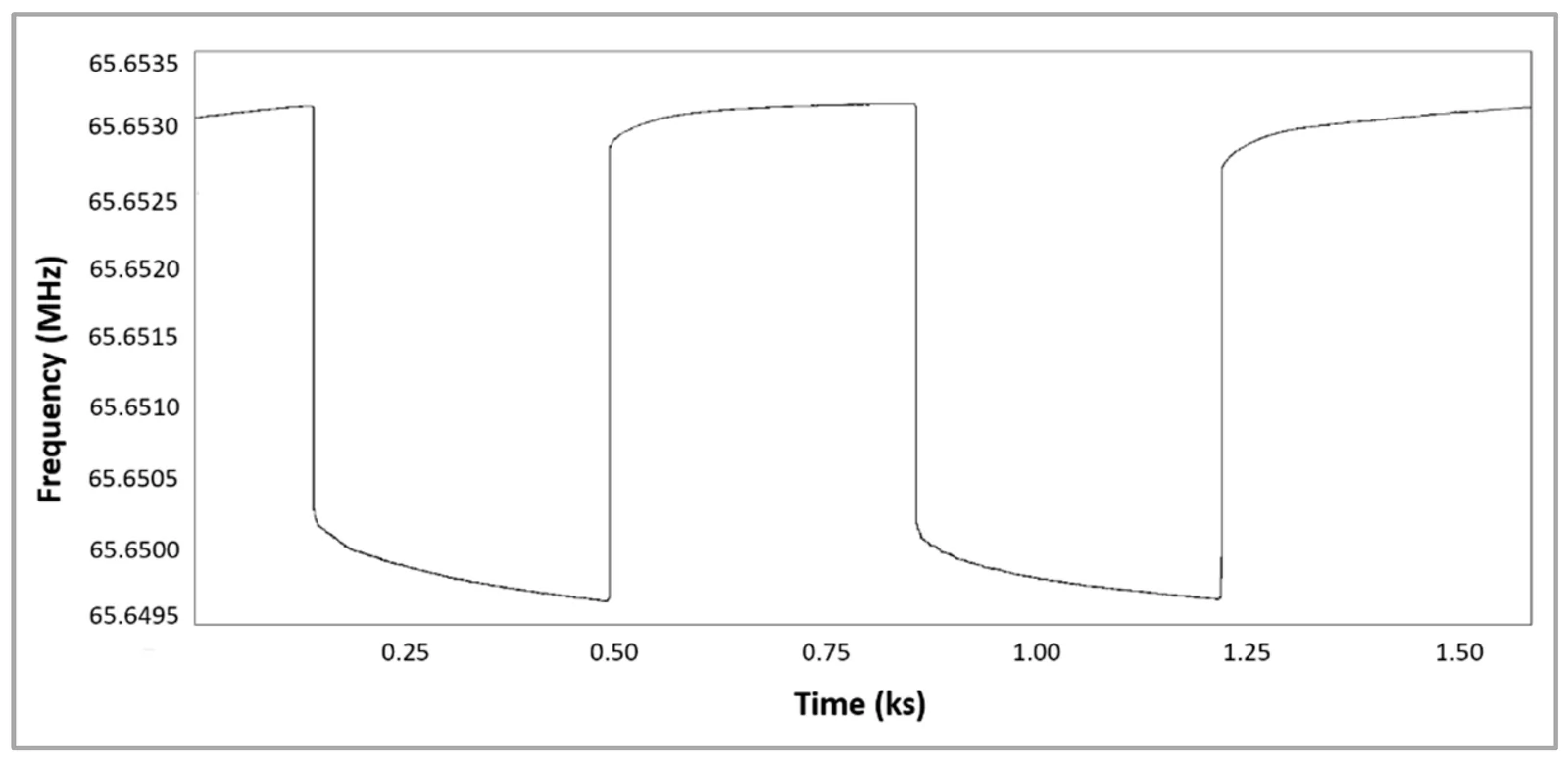
Repeatability and dynamic response of the SAW S4 sensor tested at 2% CH_4_.

**Figure 9:**
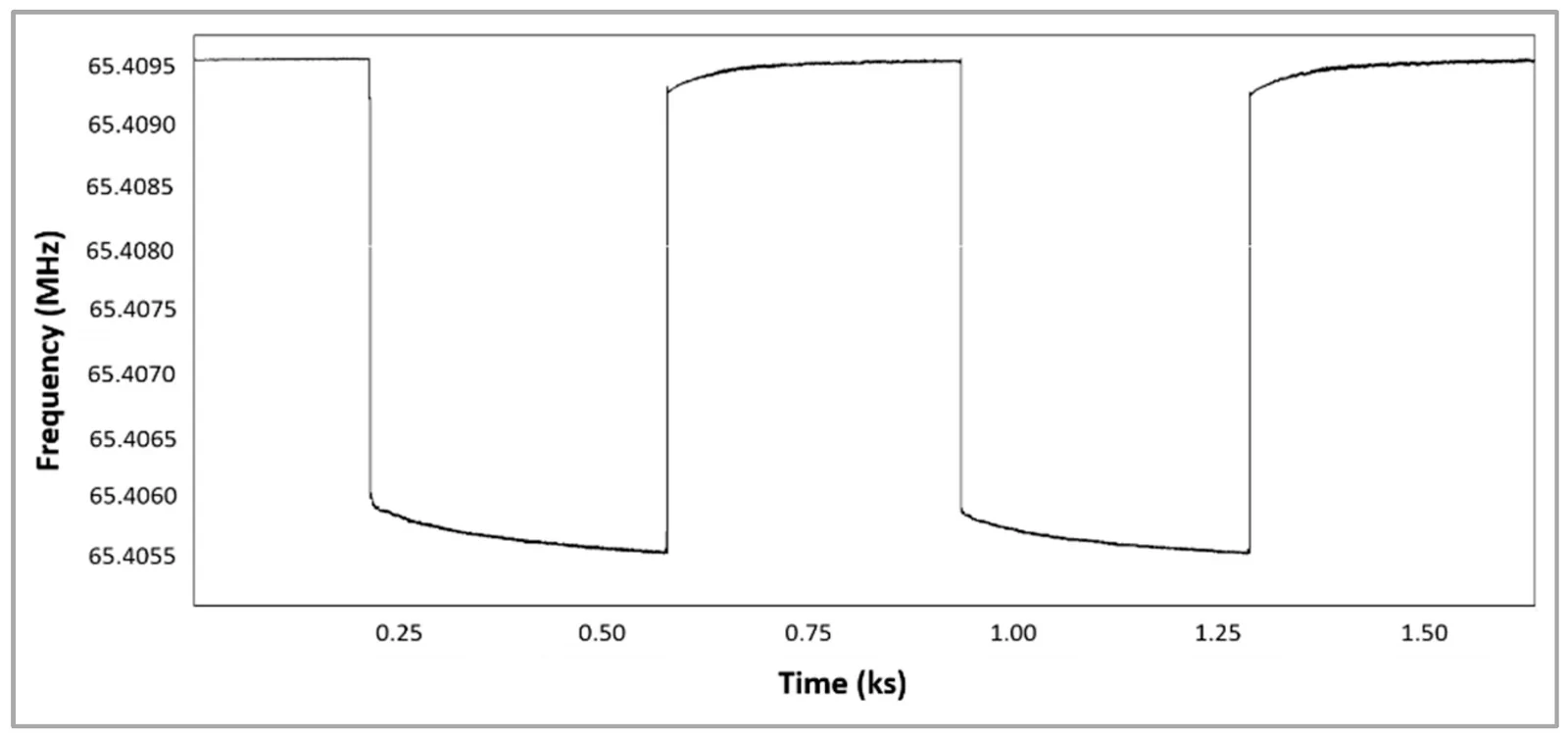
Repeatability and dynamic response of the SAW S6 sensor tested at 2% CH_4_.

**Table 1:** Sensor characteristics and film deposition pressures.

Sensor	Sensitive Material	Oxygen Pressure (mTorr)
S1	ZnO	100
S2	ZnO	700
S3	ZnO	100
	In_2_O_3_	700
S4	ZnO	700
	In_2_O_3_	700
S5	ZnO	100
	CuO	700
S6	ZnO	700
	CuO	700

**Table 2:** Sensitivity and estimated limit of detection (LOD) of the SAW sensors, calculated from the measured frequency responses to CH_4_.

Sensors	Sensitivity (Hz/ppm)	Estimated LOD (ppm)
S1	0.02	1293
S2	0.13	226
S3	0.03	871
S4	0.14	209
S5	0.05	663
S6	0.18	167

Sensitivity—(Δf/c); Δf—frequency shift; c—CH_4_ concentration; and *n*—noise level of ~10 Hz; estimated LOD—(3 × *n*)/(Δf/c).

**Table 3:** Comparison of the sensing performance of the S6 sensor with previously reported SAW-based CH_4_ sensors.

Sensitive Material	Working Temperature	Tested CH_4_ Range	LOD (ppm)	Response/Recovery Time (s)	Humidity Conditions	Reference
Cryptophane-A	20 °C	0.2–5%	~500 *	~12/20	0, 20, 30% RH investigated	[39]
Graphene/Ni/Cryptophane-A	22 °C	0–5%	NR	41.2/57	46% RH	[6]
Carbon nanowalls, H_2_ plasma-treated	RT	0.02–0.1%	4.3 *	NR	NR	[40]
W,V-doped TiO_2_/Pd NPs in PEI	RT	0.4–2%	17 *	30–60/50–90 **	NR	[41]
Monolayer graphene oxide	RT	1–20; 100–1000 ppm	1	16.7/66.4 ***	NR	[3]
ZnO/CuO	RT	0.4–2%	167 *	NR	NR	This work

NR—not reported; RH—relative humidity; LOD—limit of detection. * Estimated/calculated LOD, not necessarily the lowest experimentally tested concentration. ** Response/recovery ranges reported for the investigated sensor configurations. *** Measured at 500 ppm CH_4_.

## Data Availability

The original contributions presented in this study are included in the article. Further inquiries can be directed to the corresponding author.
